# Acknowledgement to Reviewers of *Behavioral Sciences* in 2017

**DOI:** 10.3390/bs8020018

**Published:** 2018-01-25

**Authors:** Behavioral Sciences Editorial Office

**Affiliations:** MDPI AG, St. Alban-Anlage 66, 4052 Basel, Switzerland

Peer review is an essential part in the publication process, ensuring that *Behavioral Sciences* maintains high quality standards for its published papers. In 2017, a total of 80 papers were published in the journal. Thanks to the cooperation of our reviewers, the median time to first decision was 27.5 days and the median time to publication was 59 days. The editors would like to express their sincere gratitude to the following reviewers for their time and dedication in 2017:
Ahn, Young-HyoLau, StephanAlleva, EnricoLawson, WilliamAlli, RabeenaLea, CharlieAlmenárez, FlorinaLee, JihyunAlwin, DuaneLee, JinAmendt-Lyon, NancyLee, KangAnderson, LeslieLenders, H.J.R.Antzoulatos, EvanLeyse-Wallace, RuthAssari, ShervinLilenfeld, LisaAsvat, YasminLim, Kam MingBaeza-Velasco, CarolinaLiu, YanBalakrishna, RamachandranLópez-Castro, TeresaBaldwin, Debora R.Lucic, LukaBalk, David E.Lue, FangBalzan, RyanLuthar, SuniyaBax, KARENMacLeod, RoderickBeaujean, AlexMaher, JudeBeckman, NancyMalow, Beth A.Belcher, John R.Maoz, UriBenneyworth, MichaelMarcus, Esther-LeeBhanot, SyonMari, HuhtalaBianco, AntoninoMartins, CatiaBiasutti, MicheleMartz, Camille “Kim”Bloomer, MelissaMazzetti, GretaBoccia, MaddalenaMcCown, WilliamBohutskyi, PavloMeloni, BrunoBragdon, Laura B.Miller-Rosser, KolleenBrazdil, MilanMissoni, SasaBrothers, BrittanyMøller, MetteBrowning, MatthewMondschein, AndrewBrugger, PeterMontero-Melis, GuillermoBuchanan, David R.Moores, CarlyBurke, TriciaMoulin, ChristopherCapra, SandraMożdżeń, EdytaCarpenter, Elisabeth CounselmanMunn-Chernoff, MelissaCarsley, DanaMuñoz, Mario ACerchione, RobertoNadal, MarcosChamberlain, RebeccaNagington, MauriceChung, Yang WoonNagy, MarkCicero, Theodore J.Narayan, AngelaClaid, EmilynNavarro Olivas, RaúlClutter, LynnNorcott, CandiceConnolly, MaureenNoyes, JanConnors, MichaelNussbaum, Jon F.Cooper, SimonOblad, TimothyCrawford, GregoryO’Connor, AkiraCutrona, CarolynOlde Rikkert, Marcel G. M.Davis, NoraOng, AnthonyDay, ElizabethOrmsbee, FloydDe Zárate, Margaret HillsOsorio, Daniel ColacoDean, ElizabethOverholser, JamesDevereaux, ChristinaPalmer, Jack A.Di Domenico, StefanoPark, JongsooDimitropoulos, GinaPathipati, PraneetiDinizulu, SonyaPeak, TerryDuker, Leah SteinPearce, MichelleDundes, LaurenPezoldt, KerstinDunphy, KimPiccardi, LauraEdidin, JenniferPickett, Scott M.Egger, GarryPino, MarcoEhrenberg, ShantelPoulos, ChrisElison, JeffPrice, Deborah M.Ellison, ChrisRaber, JacobEmich, Kyle J.Regolin, LuciaEnge, SörenReybrouck, MarkEvola, VitoReynolds, DeeEwert, Alan W.Rice, EricFayard, Jennifer V.Riordan, MonicaFerguson, MareeRittenour, ChristineFerrer-García, MartaRiva, GiuseppeFink, Rainer H. A.Rivera, VictorFitzgerald, TerenceRobinson, AthenaFlack, WilliamRodriguez, Francisca SFleckenstein, JohannesRose, ThedaFletcher, Barbara A. SworeRuby, PerrineFröst, PeterRussell, JamesFry, CharlotteSajnani, NishaGambetti, ElisaSalari, SoniaGangl, KatharinaSamaritter, RosemarieGebhardt, GarySamsam, MohtashemGeraghty, Keith JSapezinskiene, LaimaGermeys, InezSchiavio, AndreaGlasofer, DeborahSchrepferman, LynnGobert, DeniseScott, BritainGordon, AllisonSergaki, MaritinaGordon, Allison ScottSidhu, ManbinderGousse, YoleneSiegel, Jessica A.Granberry, PhillipSilverman, Michael JGrunseit, AnneSiniscalco, DarioGu, ZhenSivan, Abigail BGuo, ZhanSivaram, SudhaHaig, BrianSkippon, Stephen M.Haikonen, PenttiSleigh, AdrianHandelman, MikaSmith, DaleHarpin, ScottSokhadze, Estate M.Hawes, SamuelStavropoulos, KatherineHeinonen, TuulaSteidl, StephanHeinz, MelindaStuyt, Elizabeth BHines, Anika L.Swartz, Holly A.Hinz, LisaTasse, MarcHo, Roger Chun-ManTaylor, BridgetHøffding, SimonTaylor, ElizabethHorback, KristinaThiara, RaviHorowitz, MardiThomas, ElizabethHosek, Angela MThoresen, LisbethHsu, Hsun-TaTieman, JenniferHudson, DarrellTilse, CherylHuntley, EdwardTopolinski, SaschaIbarra, Irene PerezTrafimow, DavidInvitto, SaraTullis, JillianIsham, EveVan Der Schyff, DylanJames, LeonVartanian, OshinJankovic, MomciloVisi, FedericoJeremiah, RohanVonk, JenniferJohnson, AmyWarner, CatherineJohnson, Gregory S.Watkins, JuliaJohnson, John A.Weisfeld, Glenn E.Johnson, Lance A.Weller, BridgetKalsi, NavkiranWells, ChristineKastrup, BernardoWemelsfelder, FrancoiseKeeley, JaredWenos, JeanneKelly, NicholeWhite, KateKerr, DeborahWhitehead, Lisa C.Ketrow, SandraWilkinson, AnneKeyton, JoannWilshire, CarolynKibbe, AlexandraWittmann, MarcKiley-Worthington, MartheWong, Chi-kin KenchiKim, Harris Hyun-sooWoodward, Amanda T.Kindermann, HaraldWoźny, JacekKing, JulietWyant, BrianKirkland, KevinXu, LinlinKitzinger, JennyYearwood, Edilma L.Ko, CelineYih, JenniferKobiske, Karie RuekertYu, RondyKulyukin, VladimirZadeh, Rana SaghaKurt, TimothyZapolski, TamikaKwan, SanSanZhu, LinLamiell, JamesZoltan, Kekecs

